# Inhibition of NF-**κ**B by Dehydroxymethylepoxyquinomicin Suppresses Invasion and Synergistically Potentiates Temozolomide and **γ**-Radiation Cytotoxicity in Glioblastoma Cells

**DOI:** 10.1155/2013/593020

**Published:** 2013-02-21

**Authors:** M. S. Brassesco, G. M. Roberto, A. G. Morales, J. C. Oliveira, L. E. A. Delsin, J. A. Pezuk, E. T. Valera, C. G. Carlotti, E. M. Rego, H. F. de Oliveira, C. A. Scrideli, K. Umezawa, L. G. Tone

**Affiliations:** ^1^Department of Pediatrics, Faculty of Medicine of Ribeirão Preto, University of São Paulo, Brazil; ^2^Laboratório de Pediatria, Hospital das Clínicas da Faculdade de Medicina de Ribeirão Preto (USP), Bloco G, Avenida Bandeirantes, 3900 Bairro Monte Alegre, 14048-900 Ribeirão Preto, SP, Brazil; ^3^Department of Genetics, Faculty of Medicine of Ribeirão Preto, University of São Paulo, Brazil; ^4^Department of Surgery, Faculty of Medicine of Ribeirão Preto, University of São Paulo, Brazil; ^5^Department of Clinics, Faculty of Medicine of Ribeirão Preto, University of São Paulo, Brazil; ^6^Department of Applied Chemistry, Faculty of Science and Techonology, Keio University, Kanagawa 223-8522, Japan

## Abstract

Despite advances in neurosurgery and aggressive treatment with temozolomide (TMZ) and radiation, the overall survival of patients with glioblastoma (GBM) remains poor. Vast evidence has indicated that the nuclear factor NF-**κ**B is constitutively activated in cancer cells, playing key roles in growth and survival. Recently, Dehydroxymethylepoxyquinomicin (DHMEQ) has shown to be a selective NF-**κ**B inhibitor with antiproliferative properties in GBM. In the present study, the ability of DHMEQ to surmount tumor's invasive nature and therapy resistance were further explored. Corroborating results showed that DHMEQ impaired cell growth in dose- and time-dependent manners with G2/M arrest when compared with control. Clonogenicity was also significantly diminished with increased apoptosis, though necrotic cell death was also observed at comparable levels. Notably, migration and invasion were inhibited accordingly with lowered expression of invasion-related genes. Moreover, concurrent combination with TMZ synergistically inhibited cell growth in all cell lines, as determined by proliferation and caspase-3 activation assays, though in those that express O^6^-methylguanine-DNA methyltransferase, the synergistic effects were schedule dependent. Pretreatment with DHMEQ equally sensitized cells to ionizing radiation. Taken together, our results strengthen the potential usefulness of DHMEQ in future therapeutic strategies for tumors that do not respond to conventional approaches.

## 1. Introduction

Glioblastoma (GBM) is the most aggressive primary brain tumor [1]. Despite improvements in neurosurgery, radiation management, and the advent of temozolomide (TMZ), the outcome of patients remains extremely poor, with a mean life expectancy of approximately one year [2], owing to its ability to infiltrate/invade surrounding tissues and inherent radio- and chemoresistance.

Over the past decade, compelling evidence demonstrated that constitutive activation of NF-**κ**B and aberrant regulation of the signaling pathways that control its activity are involved in cancer development and progression, as well as in resistance to therapy in many types of malignancies including GBM [3–6]. Thus, inhibition of the NF-*κ*B pathway seems to be a promising option to improve the efficacy of conventional anticancer therapies.

A plethora of NF-**κ**B inhibitors has shown to be effective against various carcinomas and lymphomas (including proteasome inhibitors, IKK inhibitors, and inhibitors of IkB phosphorylation) [7], though, most of them barely discriminate between malignant and normal cells and result in unexpected side effects at required treatment doses.

 Dehydroxymethylepoxyquinomicin (DHMEQ), a synthetic compound derived from the naturally occurring antibiotic epoxyquinomicin C, has shown to possess anti-inflammatory and anticancer properties, by specifically inhibiting NF-*κ*B DNA binding, and transcriptional activity, a mechanism of action that has important clinical implications [8, 9].

Most interestingly, a recent article by Fukushima et al. [10] demonstrated that DHMEQ shows antiproliferative effects in GBM, though treatment alone was not enough to induce complete remission of xenografted tumors. Therefore, in the present study, the hypothesis that DHMEQ may decrease viability and overcome inherent chemoresistance and radioresistance of GBM cells was further investigated in a panel of 6 GBM cell lines.

## 2. Methods

### 2.1. Cell Culture

The adult human GBM cell lines T98G, U251, U138MG, and U87MG were purchased from the American Type Culture Collection, USA. The U343MG-a cell line was kindly provided by Professor James T. Rutka, (The Arthur and Sonia Labatt Brain Tumor Research Centre, Canada), while the line LN319 was a gift from Dr. Frank Furnari (Ludwig Institute for Cancer Research, CA). Cells were cultured in HAM F10 (Gibco BRL, Life Technologies, Carlsbad, CA, USA) supplemented with 10% fetal bovine serum (FBS), penicillin (100 U/mL), and streptomycin (100 *μ*g/mL) at 37°C in a humidified 5% CO_2_ incubator. Cells were fed every 2-3 days and used for the experiments until the 10th passage after thawing.

### 2.2. Chemicals and Treatments

DHMEQ was synthesized as previously described [11]. It was dissolved in dimethyl sulfoxide (DMSO) (Sigma-Aldrich, St. Louis, MO, USA) to prepare a 10 mg/mL stock solution. For combinatorial treatments temozolomide (TMZ) was purchased from Sigma-Aldrich (St. Louis, MO, USA) and diluted in DMSO according to the manufacturer's instructions. Corresponding control cultures received an equal volume of solvent (final concentration in culture medium was always less than 0.2%).

### 2.3. Cell Growth Assay

The growth-inhibitory effects of DHMEQ were determined using the XTT assay (XTT II; Roche Molecular Biochemicals, Indianapolis, IN). Briefly, equal amounts of cells were seeded in 96-well flat-bottom plates (2,000 cells/well) and allowed to grow overnight. Subsequently, cells were treated with different concentrations of DHMEQ (2.5, 5, 10, and 20 *μ*g/mL) or combinations of DHMEQ with 250 *μ*M TMZ and incubated for 24, 48 and 72 h. After treatment, the culture medium was removed and replaced with medium containing 10 *μ*L of XTT dye (3 mg/mL) in each well. The plates were incubated for 2 h at 37°C and results were interpreted by using an iMark microplate reader (Bio-Rad Laboratories, Hercules, CA, USA). Cells treated with the same concentrations of DMSO served as controls. 

### 2.4. Colony Formation Assay

Clonogenic assays were performed according to Franken et al. [12]. Single cell suspensions of 300 cells were seeded in 6-well plates and treated with DHMEQ at different concentrations for 48 h. After that period, the culture medium was replaced with drug-free medium. The cell cultures were incubated for 10 days, and then the colonies were fixed with methanol and stained with Giemsa. Only colonies with >50 cells were counted. The plating efficacy (PE) that represents the percentage of cells seeded which grow into colonies under a specific culture condition of a given cell line, was calculated as the percentage of counted colonies/seeded cells ∗ 100. The surviving fractions (SF) were determined as the number of colonies formed for a specific treatment/ number of cells seed ∗ PE [12].

### 2.5. Detection of Apoptosis

Quantification of apoptosis was determined through measurement of caspase-3 activation. Briefly, 3 × 10^4^ cells were seeded on 6-well plates containing 3 mL of culture medium. After 24 h, the medium was replaced and cells were treated with the different concentrations of DHMEQ or DHMEQ combined with TMZ (250 *μ*M) and cultured for additional 24 and 48 h. Caspase activation was determined using the NucView 488 Caspase-3 Detection in Living Cells kit (Biotium Inc. Hayward, CA, USA) according to the manufacturer's instructions. Five hundred nuclei were analyzed by fluorescence microscopy per treatment.

### 2.6. Detection of Necrotic-Like Cells

Differential staining with propidium iodide was also used to monitor cell death induction by DHMEQ after 24 and 48 h of treatment. Treated cells were simultaneously stained with bisbenzimide (Hoechst 33342), propidium iodide, and fluorescein diacetate (Sigma Chemical Co., St. Louis, MO, USA) according to Lee and Shacter [13]. Cells were analyzed by fluorescence microscopy and categorized as follows: (1) normal: blue nucleus and green cytoplasm, (2) apoptotic: fragmented blue nucleus and green cytoplasm, and (3) necrotic: red nucleus. Five hundred nuclei were analyzed per treatment.

### 2.7. Cell Cycle Analysis

For cell cycle analysis, GBM cell lines were treated with 10 *μ*g/mL of DHMEQ for 48 h. After treatment, cells were collected, fixed in 70% ethanol, stained with propidium iodide, and analyzed on a Guava Personal Cell Analysis system (Guava Technologies, Hayward, CA, USA) according to the standard protocol provided by the manufacturer. For each sample, data from 5,000 cells was recorded and percentages of cells in G_0_/G_1_, S, or G_2_/M phase were scored using the GUAVA Cytosoft 4.2.1 version Software. 

### 2.8. *In Vitro* Scratch Assay for Analysis of Cell Migration

*In vitro* scratch assays to quantify tumor migration rates were performed according to Liang et al. [14] with slight modifications. Briefly cells were grown to confluence, scratch wounds were then created using a pipet tip (200 *μ*L), and the wound site was photographed digitally at time zero. Cells were then treated with different concentrations of DHMEQ and subcultured for 24 h in medium supplemented with only 1% FBS. This low percentage of serum is used in the growth media to minimize cell proliferation and to prevent apoptosis and/or cell detachment. After that period, cells were photographed and the Motic Images Plus v2.0 software (Motic China Group Co., Ltd.) was then used to calculate the cell-free area. Cell migration rate was calculated as the distance (nanometers) travelled by the cells in this area over time.

### 2.9. Invasion Assay

5 × 10^5^ cells were treated with different concentrations of DHMEQ and transferred to the top of Matrigel-coated invasion chambers (24-well insert, 8-*μ*m pore size; Becton Dickinson & Co., NJ, USA) in a serum-free HAM-F10. Medium containing 10% fetal calf serum was added to the lower chamber as a chemoattractant. After 22 h of incubation, noninvading cells were removed from the upper surface of the membrane by scrubbing with moistened swabs. The invasive cells attached to the lower surface of the membrane insert were fixed in 100% methanol for 10 min and stained with Giemsa (Sigma-Aldrich, St. Louis, MO, USA). Membranes were then removed from the insert housing with scalpel blade, placed on a microscope slide, mounted with Entellan, and coverslipped. Invading cells were photographed under the microscope at 100x magnification and counted with the CytolabView software (Applied Spectral Imaging [ASI], Migdal Ha'Emek, Israel). 

### 2.10. Cell Irradiation

To test the effects of DHMEQ on radioresistance, clonogenic assays were performed. Single cell suspensions of 300 cells were seeded in 6-well plates and treated with DHMEQ (10 *µ*g/mL) for 24 h. After that period, the culture medium was replaced with drug-free medium, and cells were exposed to 2, 4, and 6 Gy of irradiation delivered by a *γ*-rays ^60^Cobalt source at a dose rate of about 0.47 Gy/min, using a Gammatron S-80 equipment (Siemens Medical Systems Inc., Iselin, NJ, USA) at the University Clinical Hospital (FMRP-USP). In the case of U87 cells, which do not form colonies, a proliferation-based assay was used, which is highly comparable to the clonogenic assay when the cells are allowed to undergo 6 cell divisions [15] After irradiation, the cells were plated in 96-well plates (100 microliters cell suspension, 500 cells/well), and the number of living cells was determined after 7 days by the proliferation XTT assay as described above. The radiation dose enhancement ratio (DER) by DHMEQ was calculated using the following formula: DER = (surviving fraction at an indicated dose of radiation alone)/(surviving fraction at an indicated dose of radiation + DHMEQ). Dose enhancement ratio = 1 denotes an additive radiation effect and DER > 1 a supraadditive effect as against a subadditive effect in the case of DER < 1 [16].

### 2.11. Quantitative Real-Time RT-PCR

Changes in transcriptional profiles of NF-*κ*B target genes caused by DHMEQ treatment (5 and 10 *μ*g/mL) were analyzed by quantitative PCR. After treatment, cells were collected, and total RNA was isolated using the Trizol Reagent (Invitrogen, São Paulo, Brazil) following the manufacturer's instructions. Reverse transcription was carried out at 37°C for 120 min with a High-Capacity kit (Applied Biosystems, Foster City, CA, EUA). Real-time RT-PCR reactions were performed in triplicate in 10 *μ*L reactions using the inventoried TaqMan probes (Applied Biosystems, Foster City, CA, EUA) for *BCL2* (Hs00608023_m1), *BCL-XL *(Hs00236329_m1), *XIAP* (Hs01597783_m1), *MMP-2 *(Hs01548727_m1), *MMP-14 *(Hs00237119_m1), *uPA* (Hs01547054_m1), *TIMP-2 *(Hs00234278_m1), and *MGMT* (Hs01037698_m1), on the ABI Prism 7500 Sequence Detector (Applied Biosystems, Foster City, CA, EUA). As endogenous controls hypoxanthine, guanine phosphoribosyltransferase (4310890E0) and TATA-binding protein (4310891E) (Applied Biosystems, Foster City, CA, EUA) were used. A pool of five white matter samples was used as a calibrator. The relative quantification was performed by the 2^−ΔΔCT^ method [17].

### 2.12. *MGMT* Promoter Methylation-Specific PCR

*MGMT* promoter methylation status was determined in GBM cell lines by using methylation-specific PCR as described [18]. A total of 1 *µ*g of genomic DNA was chemically modified by sodium bisulfite [19]. Two PCRs reactions were performed in each sample, one to detect methylated *MGMT* promoter sequences(5′-GTTTTTAGAACGTTTTGCGTTTCGAC-3′ and 5′-CACCGTCCCGAAAAAAAACTCCG-3′) and, other to detect unmethylated *MGMT* promoter sequences (5′-TGTGTTTTTAGAATGTTTTGTGTTTTGAT-3′ and 5′-CTACCACCATCCCAAAAAAAAACTCCA-3′) [20]. Each PCR product was separated on 2% agarose gels. As positive control sample, we used genomic DNA from U87 glioma cell line, which carries a completely methylated *MGMT* promoter. Genomic DNA extracted from peripheral blood leukocytes treated with 5-aza-2′-deoxycytidine (decitabine) served as unmethylated control sample. In addition, a control reaction without any template DNA was performed together with each PCR experiment.

### 2.13. Comet Assay

In order to further test the ability of DHMEQ to enhance TMZ-induced DNA damage, single cell gel electrophoresis assay (comet assay) was performed as described previously by Singh et al. [21]. Briefly, 5 × 10^4^ cells (T98G and U138MG) were seeded in 6-well plates and incubated for 24 h. After that period cells were pretreated with DHMEQ 10 *μ*g/mL for 6 h and then exposed to TMZ (250 *μ*M) and reincubated for further 6 h. Consequently, the cultures were washed with PBS solution and trypsinized. Single-cell suspensions were centrifuged for 5 min (500 rpm) at 4°C. The pellet was resuspended in 100 *μ*L of 0.5% (w/v) low-melting point agarose (Gibco, Carlsbad, CA, USA) and the mixture spread onto two microscope slides precoated with 1.5% (w/v) normal-melting point agarose (Gibco, Carlsbad, CA, USA) and coverslipped. When the gels had solidified, the coverslips were gently removed and the slides were immersed in cold (4°C) lysis solution (1% Triton X-100, 10% DMSO, 2.5 mM NaCl, 100 mM Na_2_EDTA, 100 mM Tris, and pH 10) for 24 h. Immediately after this step, slides were placed in a horizontal electrophoresis unit containing freshly prepared electrophoresis buffer (1 mM Na_2_EDTA, 300 mM NaOH, and pH > 13). The DNA was allowed to unwind for 20 min, and subsequently electrophoresis was performed at 25 V, 300 mA for 20 min. The slides were then gently immersed in a neutralization buffer (0.4 M Tris-HCl, pH 7.5) for 15 min and fixed with ethanol. Before analysis slides were stained with 20 *μ*L/mL SYBR Green (Invitrogen, Carlsbad, CA, USA), images of 50 nucleoids per slide were captured under a Zeiss fluorescent microscope equipped with an excitation filter of 515–560 nm and a barrier filter of 590 nm (409 objective) and digital fluorescent images were obtained using the AxioVision 3.1 software (Zeiss, Gottingen, Germany). The relative length and intensity of DNA tails to heads were proportional to the amount of DNA damage present in the individual nucleus and were measured by Olive tail moment with TriTek Comet Score software (TriTek, Sumerduck, VA, USA).

### 2.14. Statistical Analysis

Statistical analyses were performed by using the SigmaStat software (Jandel Scientific Company, San Rafael, CA, USA). Two-Way Repeated Measures Analysis of Variance (ANOVA) followed by the Holm-Sidak Pairwise Multiple Comparison was used to establish whether significant differences existed between groups. All tests were carried out for *α* = 0.05. Effective concentrations (IC_50_) were analyzed using the CalcuSyn software v2.0 (Biosoft, Ferguson, MO). This program provides a measure of the combined drug interaction by the generation of a combination index (CI) value. The CI value is based on the multiple drug-effect equation of Chou and Talalay (1984) and defines the drug interactions as synergistic CI value <1, additive CI value =1, or antagonistic CI value >1. Calcusyn software was also used to calculate the dose reduction index (DRI) of drug combinations which estimates the extent to which the dose of one or more agents in the combination can be reduced to achieve effect levels that are comparable with those achieved with single agents [22].

## 3. Results 

### 3.1. DHMEQ Inhibits Growth in GBM Cells

**Figure 1:**
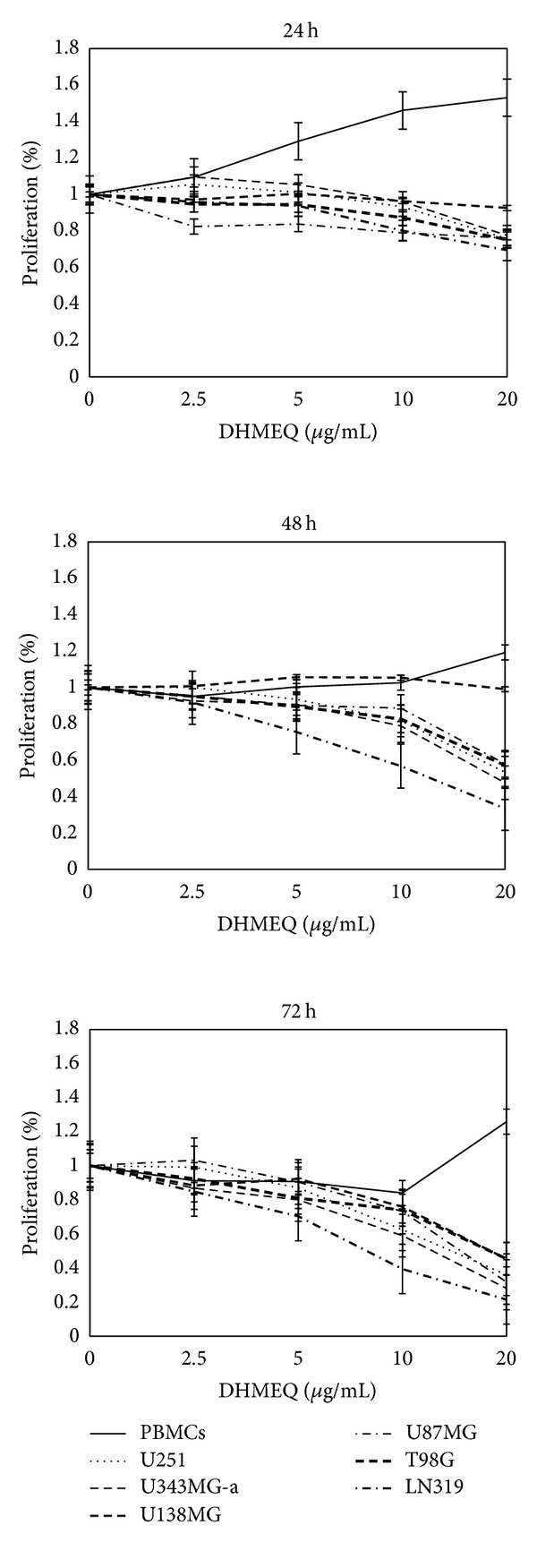
DHMEQ significantly reduced cell proliferation in time- and dose-dependent manners in all GBM cell lines; such effects were not observed in peripheral blood mononuclear cells (PBMCs) confirming once again the high selectivity of this drug. Cells were exposed to different concentrations of DHMEQ (2.5 to 20 *μ*g/mL) and analyzed by using the XTT assay after 24, 48, and 72 h. All experiments were performed three times in triplicate. Error bars: mean ± SD.

Differentially from previously reported results [10], in the present study all the 6 cell lines tested were sensitive to DHMEQ treatment. Results of XTT assays showed growth inhibitory effects of DHMEQ in dose- and time-dependent manners when compared to the vehicle control (DMSO). After 24 h of treatment, statistically significant results (*P* < 0.05) were only observed for cells treated with 20 *μ*g/mL; at longer periods, statistical differences were obtained after treatments with 5, 10, and 20 *μ*g/mL (*P* < 0.05). U138MG cells were more resistant to DHMEQ though resistance was circumvented after a longer period of exposure (72 h), reducing growth in 55% (at 20 *μ*g/mL) compared to control. Treatment of the other 5 cell lines with 20 *μ*g/mL of DHMEQ for 72 h also elicited a marked inhibition of growth by 64% in U251, 72% in U343MG-a, 69% in U87MG, 56% in T98G, and 79% in LN319 (Figure 1). Concentrations required to cause 50% cell growth inhibition (IC_50_) varied between cell lines (Table 1). Mean IC_50_ was calculated as approximately 26 *μ*g/mL and 14 *μ*g/mL after 48 h and 72 h of treatment, respectively. 

### 3.2. DHMEQ Induces Cell Death in GBM Cells

**Figure 2:**
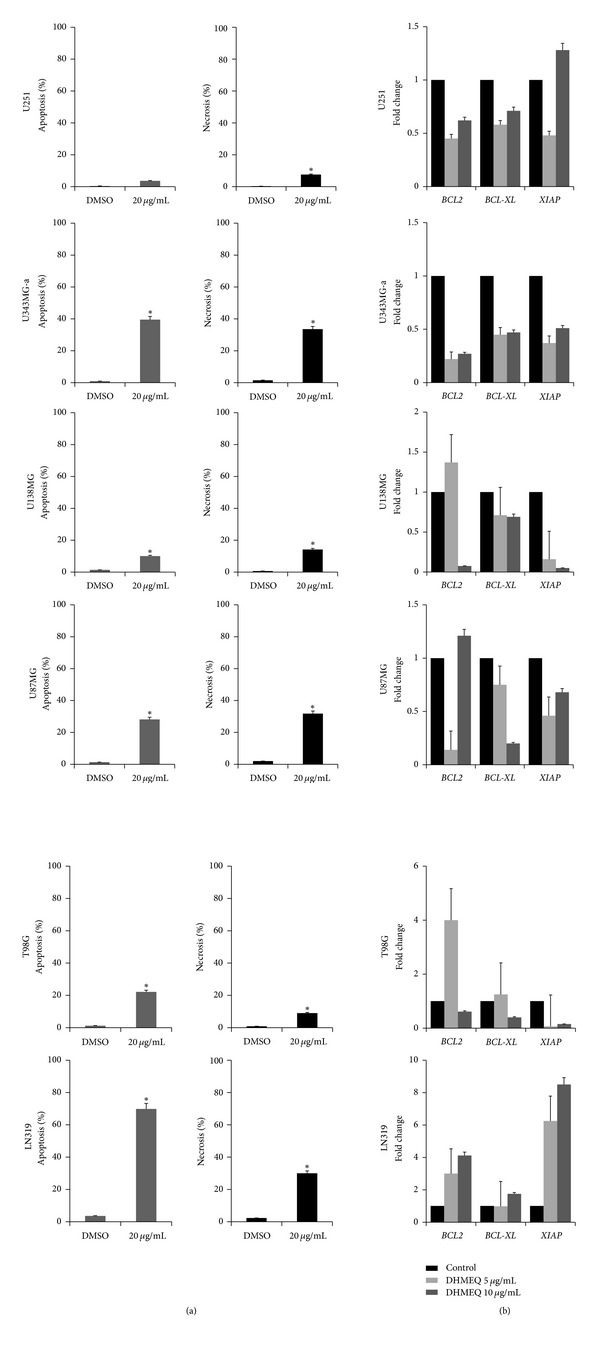
(a) Treatment with DHMEQ (20 *µ*g/mL) significantly increased apoptosis in 5 out of 6 cell lines. Cells were treated for 48 h and caspase-3 activation (Nucview 488 kit) was measured by fluorescent microscopy. Comparable levels of necrosis-like cell death (detected by differential staining with propidium iodide) were observed after treatment with the same dose after the same period. Data represents three independent experiments and are expressed as mean ± SD (*P < 0.05). (b) Human GBM cells were exposed to DHMEQ (5 and 10 *μ*g/mL) for 24 h at which time RNA was collected and used for qRT-PCR for the apoptosis-related genes *BCL-XL, BCL2,* and *XIAP*. Data represents two independent experiments in duplicate and are expressed as mean ± SEM.

Induction of cell death by DHMEQ was evaluated by two different methodologies (caspase-3 activation and differential staining with propidium iodide) and analyzed by fluorescence microscopy. Treatment of cells for 48 h with DHMEQ caused a significant increase in the levels of caspase-3 activity in five out of six cell lines when compared to untreated controls, though such increase was only observed after treatment with 20 *μ*g/mL (Figure 2(a)) and was not time dependent. U251 was resistant to treatment but showed a 15% increase of apoptosis after a longer period (72 h) of treatment (data not shown). Comparatively, DHMEQ also triggered necrosis-like cell death after treatment with the same concentration at 48 h. The more sensitive cell lines were U343MG-a and LN319 with almost 90% of cell death when both methodologies were considered (Figure 2(a)). 

Changes in the expression levels of antiapoptosis genes regulated by NF-*κ*B were also investigated after treatment with DHMEQ. Decreased expression of *BCL2* was observed in U251 and U343MG-a at both concentrations tested and for U138 MG and T98G after treatment with 10 *μ*g/mL. Downregulation of *BCL*-XL, was observed in 5 out of 6 cell lines, though for T98G overexpression was observed after 5 *μ*g/mL treatment, whereas the expression levels of the anti-apoptotic gene *XIAP *were decreased in 4 cell lines (U251 showed reduced levels only after treatment with the lowest concentration). Contrary to expected, LN319 showed upregulation of all three transcripts despite being the most sensitive to treatment with DHMEQ (Figure 2(b)).

### 3.3. DHMEQ Induces Cell Cycle Arrest

**Figure 3:**
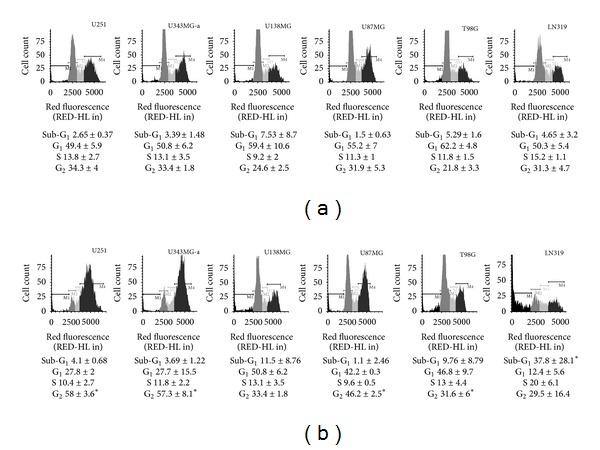
Cell cycle analysis after treatment with DHMEQ (10 *μ*g/mL) for 48 h. Cells were collected, stained with propidium iodide, and analyzed on a Guava Personal Cell Analysis system according to the standard protocol. When compared to controls (a), DHMEQ exerts arrest in G_2_/M phase of cell cycle (b) (*P < 0.05). Percentages of cells in G_1_, S, and G_2_/M phases are expressed as mean ± standard deviation of at least 3 independent experiments.

Treatment with DHMEQ (10 *μ*g/mL) induced a prominent and sustained change in the cell cycle distribution. As shown in Figure 3, within 48 h treated cells significantly accumulated in the G_2_/M phase in 4 out of 6 cells. The percentage of the cells in G_1_ and S phases decreased in the same proportion as a result of treatment while untreated cells (control) were more evenly distributed throughout the cell cycle. U138MG cells showed a moderate increase in the G_2_/M population. In the case of LN319, a great proportion (about 40%) of cells in Sub-G_1_ was observed, coincident with the high number of necrotic-like cells observed under the microscope for cell death testing.

### 3.4. DHMEQ Potently Abrogates the Clonogenic Capacity of GBM Cell Lines

**Figure 4:**
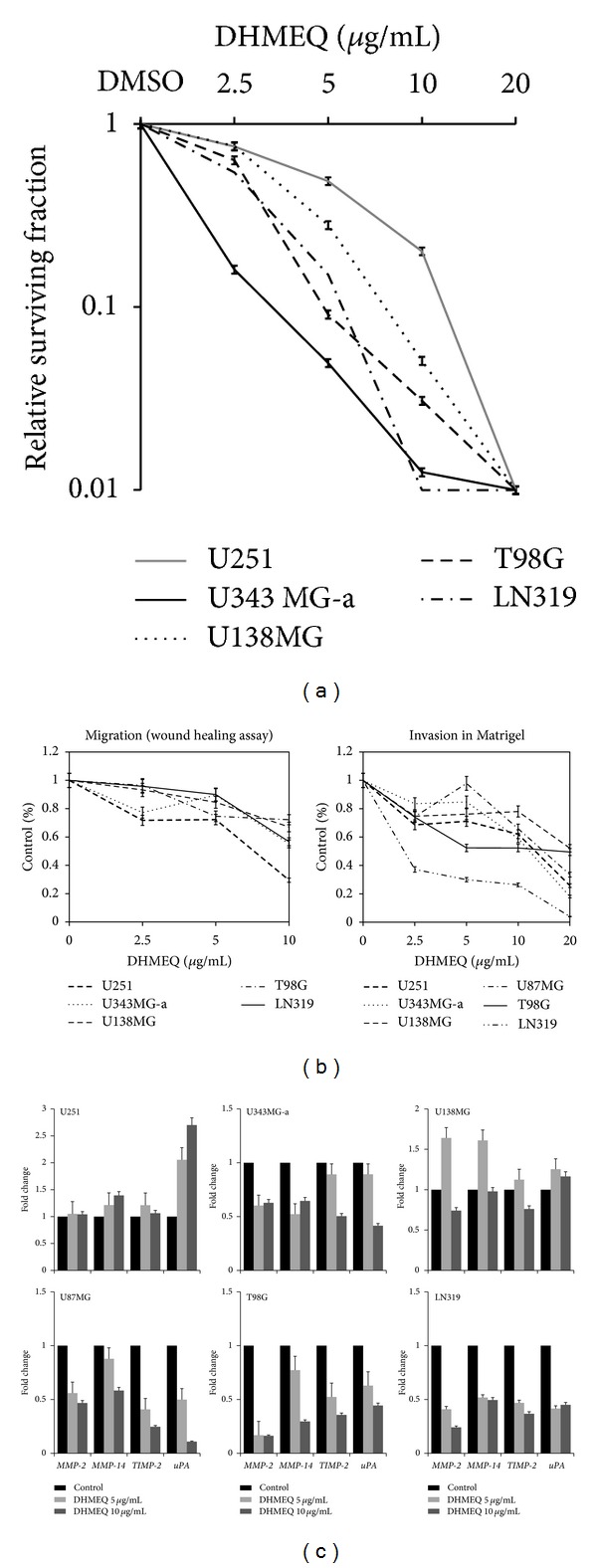
(a) DHMEQ potently abrogated the retained capacity for producing progeny of all GBM cell lines as detected by the clonogenic assay after 48 h of treatment. Each value represents the mean derived from at least three individual experiments (mean ± SD); (b) all cell lines presented a significant decrease in invasion potential as demonstrated by wound healing assay (significant after treatment with 10 *μ*g/mL) and Matrigel-coated chambers (significant at all concentrations tested). Each value represents the mean derived from at least three individual experiments (mean ± SD); (c) migration-associated gene expression was also diminished in (4 out of 6) human GBM cells. In this case, cells were analyzed after treatment with 5 and 10 *μ*g/mL of DHMEQ for 24 h in the presence of 1% FBS. Results are presented as normalized relative expression levels compared with control (DMSO) samples. Data represents two independent experiments in duplicate and are expressed as mean ± SEM.

NF-*κ*B inhibition by DHMEQ significantly reduced the colony formation capacity for all cell lines when compared to control (*P* < 0.05) at all concentrations tested (Figure 4(a)), demonstrating long-term effects even after removal of the drug. Mean reductions were calculated as 43% (ranging from 25 to 84%), 78% (ranging from 53 to 93%), and 94% (ranging from 80 to 99%) after treatment with 2.5, 5 and 10 *μ*g/mL, respectively. No countable colonies were observed when cells were exposed to 20 *μ*g/mL of DHMEQ. 

### 3.5. DHMEQ Inhibits Cell Migration and Invasion *In Vitro*

DHMEQ significantly reduced cell migration as measured by *in vitro* wound healing assays at the highest concentration tested (10 *μ*g/mL) for all cell lines (*P* < 0.05) (Figure 4(b)). Invasion assay using transwell chambers coated with Matrigel showed significant reductions of invasion at all concentrations tested for all cell lines (except for U87MG at 5 *μ*g/mL) in a dose-dependent manner. Maximum reductions in invasive potential were calculated as 75% for U251, 82% for U343MG-a, 48% for U138MG, 67% for U87MG, 51% for T98G, and 97% for LN319 (Figure 4(b)).

Treatment with the NF-*κ*B inhibitor also reduced the mRNA levels of *MMP-2 *in U343MG-a, U87MG, T98G, and LN319. Expression levels of this metalloproteinase for U138MG were only observed with the highest concentration. Similarly *MMP-14 *expression showed reductions in U343MG-a, U87MG, T98G, and LN319 cells while in U138MG expression of this gene was observed only after 10 *μ*g/mL treatment.* TIMP-2 *and *uPA* expression levels were also diminished in a dose-dependent manner in 4 out of 6 cell lines, after treatment with 10 *μ*g/mL for 24 h in the presence of only 1% FBS (Figure 4(c)). Expression levels of these four transcripts were not affected or were upregulated in U251.

### 3.6. DHMEQ Shows Potent Synergistic Effects When Combined with TMZ

**Figure 5:**
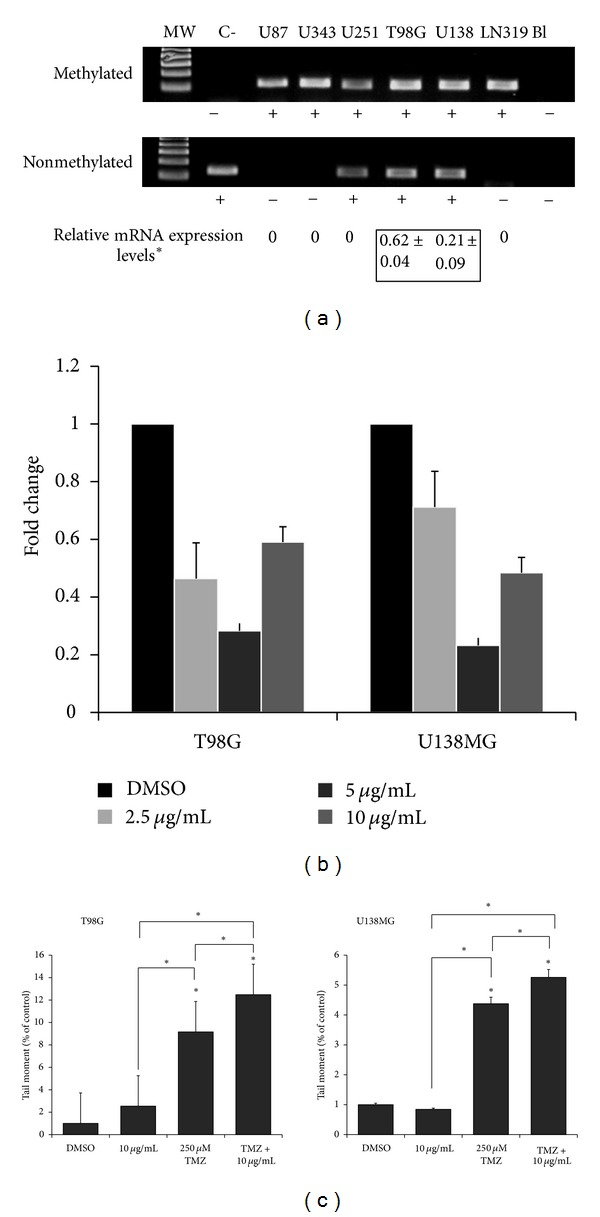
(a) The analysis of the methylation status of the *MGMT* gene in GBM cell lines revealed two groups: methylated (U87MG, U343MG-a, and LN319) and hemimethylated (U251, T98G and U138MG); however, only T98G and U138MG express this gene as detected by real time quantitative PCR (*a pool of 5 white matter samples was used as calibrator); (b) treatment with DHMEQ efficiently decreases the expression of MGMT after 24 h. Data represents two independent experiments in duplicate and are expressed as mean ± SEM; (c) comet assay showed that TMZ-induced DNA damage significantly increases in T98G and U138MG cells as a probable consequence of reduced MGMT expression after exposure to DHMEQ. Each value represents the mean derived from at least three individual experiments (mean ± SD).

In order to test whether DHMEQ can enhance the cytotoxic effects of TMZ, the major chemotherapeutic agent used in the treatment of patients with GBM, we first determined the methylation status of the promoter and expression levels of the *MGMT* gene. As seen in Figure 4(a), half of cell lines (U87MG, U343MG-a, and LN319) presented methylated *MGMT* promoters, while in the rest (U251, T98G, and U138MG), the promoters were hemimethylated. Despite this, only T98G and U138MG showed *MGMT* expression as detected by quantitative PCR (Figure 5(a)). Cellular sensitivity of each of the six cell lines to TMZ was also evaluated after 48 and 72 h of treatment. IC_50_ values varied between the different cell lines (Table 1). Based on this data, cell lines were considered as sensitive (U251, U343MG-a, U87MG, and LN319) or resistant (T98G and U138MG) to TMZ. For combination with DHMEQ, the dose of 250 *μ*M TMZ was selected, which inhibited growth in about 25–30% in the sensitive cell lines but had no growth inhibitory effects on T98G and U138MG. 

Drug interaction testing between DHMEQ and TMZ was performed using nonlinear regression of a sigmoid dose response model and combination index (CI) approaches. The results revealed synergistic effects (CI < 1) when cells were simultaneously treated at all concentrations of DHMEQ for U343MG-a, U87MG and LN319. For U251 synergistic effects were only observed after simultaneous treatment with 20 *μ*g/mL and TMZ 250 *μ*M. For cells resistant to the latter (T98G and U138) only antagonistic effects were observed at all treatments (Table 2). However, this response was drastically reversed when the administration schedule was changed. Pretreatment with the different concentrations of DHMEQ for 6 h before exposing cells to TMZ, sizably sensitized all cell lines. Sequential exposure also resulted in high DRI values suggesting that TMZ doses could be significantly reduced to achieve comparable cytotoxicity (Table 2). Schedule dependency might be in part a result of the inhibition of the transcriptional regulation of MGMT. As seen in Figure 5(b), the expression of this gene was significantly reduced after treatment with DHMEQ, even at low concentrations. Moreover, reduced MGMT activity was also indirectly evinced after evaluating the degree of DNA damage exerted by TMZ with the previous (6 h) exposure to the NF-*κ*B inhibitor (Figure 5(c)).

**Figure 6:**
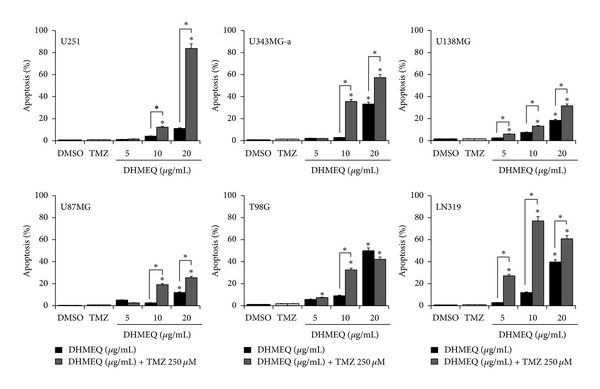
Compared to single treatments, associations of DHMEQ and TMZ synergistically increase apoptosis rates in all cell lines tested after 24 h (*P < 0.05). Each value represents the mean derived from at least three individual experiments (mean ± SD).

Synergism between both drugs was also addressed through caspase-3 activation. TMZ alone at 250 *μ*M did not show any apoptosis induction for all cell lines compared with control. Differently, when combined with DHMEQ apoptosis rates were significantly (*P* < 0.05) increased compared to single treatments, even at lower doses of DHMEQ (Figure 6). Interestingly, the U251 cell line, which had shown to be insensitive to DHMEQ (or to TMZ at 250 *μ*M), showed more than 80% of apoptotic cells when both drugs were combined. 

### 3.7. DHMEQ Pretreatment Sensitizes Cells to Ionizing Radiation

**Figure 7:**
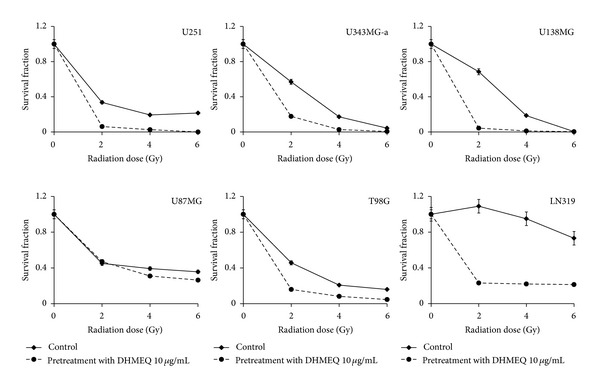
Clonogenic survival assay of GBM cell lines irradiated with 2, 4 and 6 Gy (control) and cells pre-treated with 10 *μ*g/mL DHMEQ for 24 h before irradiation with the same doses. Treatment significantly radio-sensitized cells. Each value represents the mean derived from at least three individual experiments in duplicate (mean ± SD).

To study the cytotoxic effects of DHMEQ in association with *γ*-radiation, single-cell suspensions of cells (clonogenic assay) were incubated with 10 *μ*M concentration of the drug for 24 h to induce G_2_ arrest. After treatment, the cell culture medium was replaced, and cells were irradiated with final doses of 2, 4, and 6 Gy. For U87MG cells, proliferation assays were applied. The results showed that DHMEQ pretreatment efficiently led to radiosensitization in all GBM cell lines (Figure 7), resulting in high dose enhancement ratios (Table 3). U251 showed complete abrogation of the colony formation capacity after combined treatment with 6 Gy.

## 4. Discussion

The limited treatment options for GBM have long motivated an exhaustive search for developing more rational and effective therapies to target molecules that support the maintenance and growth of the tumor cells. Like in most human cancers, deregulation of the NF-*κ*B pathway promotes GBM tumor growth and progression through the transcriptional activation of genes associated with suppression of apoptosis, metastasis and resistance to cytotoxic agents [23]. Thus far, more than 800 therapeutic compounds that inhibit either activation or function of NF-*κ*B have been identified, including a variety of natural and synthetic molecules [7] which in turn have shown growth inhibitory effects in this high grade tumor [24–29].

In the present study, we analyzed the effects of NF-*κ*B inhibition by DHMEQ on the survival and chemo/radioresistance of six adult glioblastoma cell lines (U251, U343MG-a, U138MG, U87MG, T98G, and LN319). The antitumor effects of DHMEQ have been continually reported *in vitro* and in *in vivo* models [30–37]. Compared to other NF-*κ*B inhibitors, this drug is distinctive by covalently binding to the highly conserved Cys38 of the Rel family members (p65, cRel, RelB, and p50), a residue that is essential for NF-*κ*B DNA-binding and transcriptional activity [8].

Our results showed that the pharmacologic inhibition of NF-*κ*B by DHMEQ in GBM cells significantly reduced cell growth (all cell lines were equally sensitive after 72 h) and prompted G_2_/M arrest in dose- and time-dependent manners while inducing apoptosis through caspase-dependent pathways. However, at doses necessary to cause apoptotic death, comparable levels of necrosis were also observed in our study. These results are compatible with previous reports which demonstrated that increasing DHMEQ concentrations change the mechanism of its cytotoxic effects from apoptosis to caspase-independent necrotic-like cell death in thyroid cells [31].

DHMEQ-induced apoptosis of GBM cells was accompanied by a down-regulation of genes involved in antiapoptosis, though the expression levels of *BCL*-*XL*, *BCL2,* and *XIAP* were not equally diminished for all cell lines. Similar results were observed by others in multiple myeloma, hepatoma, and Burkitt lymphoma [38–40]. Even though NF-*κ*B is a pleiotropic transcription factor that simultaneously regulates multiple targets, cumulative evidence demonstrates that the promoter/enhancer regions of most genes contain more than one transcription factor response element so there is a frequent crosstalk between NF-*κ*B and an ever-expanding list of other transcriptional regulators (such as STAT3, HIF-1a, AP1, SP, TP53, PPARc, and *β*-catenin) that might alter gene expression in different ways [41]. Other factors might also contribute to determine the proper regulation of genes, such as chromatin structure and epigenetic state of target genes, as well as the individual Rel proteins that form NF-*κ*B homodimers and/or heterodimers [42, 43]. Consequently the dependence of the gene expression on NF-*κ*B may be different in each GBM cell line. A clear example comes from the observed dissimilar behaviors between LN319 and U251. Most certainly, both cell lines differ in numerous genetic alterations and U251 cells, which showed downregulation of *BCL2* and *BCL-XL,* may no longer solely depend on NF-*κ*B activity for survival. LN319, on the other hand, was highly sensitive to treatment but showed upregulation of all three antiapoptotic genes. Nonetheless, *BCL-XL* is a classic anti-apoptotic gene whose inactivation has shown to be sufficient to trigger apoptosis in U87MG and GBM primary cultures [44].

On the other hand, DHMEQ treatment was able to reduce in dose-dependent manner the expression of the metastasis-promoting genes *MMP-2, MMP-14, TIMP-2,* and *uPA*, all of which have been implicated in the regulation of invasion in glioma cells [45–48]. The ability of cancer cells to metastasize requires a succession of orchestrated molecular events that include loss of cell-cell adhesion, migration through blood vessels, and establishing growth at a distant site. Even though GBM does not tend to metastasize outside the brain, the ability of tumor cells to invade surrounding tissues is a forefront problem that hampers tumor resection and remains the main cause of local disease recurrence, and death of patients with primary tumors [49]. Our results showed that treatment with DHMEQ limits every essential step in the metastasis process: it reduces the expression of matrix-degrading proteases, functionally mitigates tumor cell spread as detected by wound healing and invasion on Matrigel assays and impairs the ability of single cells to form colonies in all six cell lines tested.

Other equally contributing factors that lead to a poor clinical response and patient outcome in GBM are inherent chemo- and radioresistance [50]. The intimate involvement of NF-*κ*B in anticancer drug resistance has been described in various *in vitro* and *in vivo* models [51]; thereby its inhibition could also potentially lead to the reversal of chemoresistance. 

For many years, fruitless attempts to improve the dismal prognosis of patients with glioblastoma included changes to radiotherapy schedule doses and techniques. In the late 1990s, the inclusion of TMZ at initial treatment seemed promising and showed to improve survival compared with radiotherapy alone in adult cohort patients [2]. TMZ is a prodrug that transforms under physiologic conditions into an active unstable methylating metabolite that results in O^6^-methylguanine adducts, ensuing DNA double-strand breakage and eventually cell death [52]. Nevertheless, most primary brain tumors express O^6^-methylguanine-DNA methyltransferase (MGMT), a cytoprotective DNA repair protein that efficiently reverses the cytotoxic effects of alkylating agents, contributing with tumor resistance [53]. As a result, MGMT expression is directly correlated with drug responsiveness and is clinically used to develop tailored treatment regimens in GBM [54].

In the present study, concomitant combinations with DHMEQ synergistically enhanced the cellular sensitivity of GBM cells to TMZ, as determined by cell proliferation and caspase-3 activation assays. Accumulating body of evidence shows that the majority of sensitizing agents exert their activity by derestricting the central apoptotic program to be “responsive” to apoptotic stimuli. Consequently, downregulation of the anti-apoptotic gene products *BCL2/BCL-XL/XIAP* via NF-*κ*B inhibition may allow tumor cells to be sensitive to apoptosis induction by low doses of TMZ, as is the case for U251 showing strongly potentiated apoptosis despite being resistant to single treatments. These results are consistent with recent studies by other investigators demonstrating that low concentrations of DHMEQ can enhance the sensitivity of tumor cells to several cytotoxic agents [34, 55–59]. However, the beneficial effects of DHMEQ were not observed in MGMT-expressing cells (T98G and U138MG), for which combined treatment highly ineffective (CI values >1). NF-*κ*B is a key regulator of MGMT transcription [60, 61] nonetheless, immediate response to TMZ clearly depends on the preexisting levels of this protein in the cell. Thus, DHMEQ needed to be administrated before cells were exposed to TMZ. This may be an important observation when designing *in vivo* preclinical models or clinical trials, since drug delivery will need to respect specific time frames. Indeed, the sequential schedule of drug administration was efficient in overcoming TMZ-resistance in both cell lines. Even a short (6 h) pretreatment with low concentrations of the NF-*κ*B inhibitor exceedingly potentiated the cytotoxic effects of TMZ, with such increased synergism also achieved in U251 (which according to the literature also shows slight levels of MGMT), U343MG-a, U138MG, and U87MG. Even though TMZ has shown to have reduced toxicity in normal cells [62], these findings demonstrate that both drugs doses could be significantly reduced (as denoted by high DRI values) to achieve comparable results in a schedule-dependent manner, what could be clinically advantageous for tumors with intrinsic or acquired drug resistance.

Additionally, pretreatment with DHMEQ (10 *μ*g/mL) highly sensitized GBM cells to low doses of ionizing radiation. Several studies have explored the impact of NF-*κ*B inhibition in various models [6, 25, 63–66] conversely, as far as we know, this is the first study to investigate the radiosensitizing potential of DHMEQ. Radiotherapy is a standard treatment for patients with GBM (Minniti et al., 2009) however, its efficiency is restricted by toxic side effects that limit dose escalation. Of note, while most of the cell lines tested is highly radioresistant [67], comparable results were obtained irrespective of TP53 mutational status which is known to contribute to differential responsiveness of GBM cells to radiation [68].

Our results indicate that DHMEQ may decrease the apoptotic threshold of GBM cells through the transcriptional block of *BCL2*, *BCL-XL*, and *XIAP*, all of which may contribute to cytoprotection. Moreover, DHMEQ, like TMZ, induces an arrest in G_2_/M which is known to be the most radiosensitive phase of the cell cycle [69].

In general, GBM is considered to be a heterogeneous group of tumors, resulting in large number of altered genes [1, 70]. However, this complexity is reduced significantly by considering the biological pathways, rather than the altered gene themselves. Persistent NF-*κ*B activity can be a result of either chromosomal amplification, overexpression, constitutive activation of upstream signaling kinases, or mutations inactivating its cytosolic inhibitors. Compared to other NF-*κ*B inhibitors, DHMEQ is unique because it directly inhibits its transcriptional ability and hence, hinders both the canonical and noncanonical pathways of activation. Although additional studies are required to determine the pharmacokinetics and safety of DHMEQ in humans, our findings along with those previously reported [10] suggest that the anti-proliferative effects and the reversal of chemo- and radioresistance by DHMEQ might have significant clinical implications. These observations place DHMEQ as an interesting compound to be tested in preclinical models and hopefully increase survival rates in patients with GBM.

## Figures and Tables

**Table 1:** Doses required to induce 50% inhibition of cell growth (IC_50_) in GBM cell lines treated with DHMEQ and TMZ.

Cell line	IC_50_ values calculated at 48 h		IC_50_ values calculated at 72 h	
	DHMEQ *μ*g/mL	TMZ *μ*M	DHMEQ *μ*g/mL	TMZ *μ*M
U251	21.78	744.51	13.50	646.18
U343MG-a	22.62	570.13	11.52	508.14
U138MG	1068.01	1315.33	17.93	1170.32
U87MG	35.06	1328.06	14.45	163.56
T98G	28.19	4106.53	18.62	1382.06
LN319	22.62	1729.74	8.00	897.31

**Table 2:** Median dose effect analysis was also employed to characterize the interactions between DHMEQ and TMZ. For concomitant treatment, different doses of DHMEQ were combined with 250 *μ*M TMZ and treated for 48 h. For the sequential schedule cells were exposed to different concentrations of DHMEQ, and after 6 h TMZ at the same dose was included in culture media and also analyzed at 48 h (TMZ exposure 42 h). Combination index (CI) values <1 correspond to a synergistic interaction (emphasized in bold). Dose reduction index (DRI) reflects the fold reduction in the required concentration of tested agents when used in combination to achieve the comparable affected fraction (AF).

U251		Concomitant TMZ (250 *μ*M)			Sequential TMZ (250 *μ*M)		
DHMEQ (*μ*g/mL)	AF	AF	CI	DRI	AF	CI	DRI
2.5	0.00	0.15	2.02	0.58	0.38	**0.68**	1.87
5	0.07	0.26	1.31	1.11	0.42	**0.73**	2.19
10	0.19	0.36	1.21	1.73	0.6	**0.59**	4.36
20	0.47	0.65	**0.83**	5.33	0.8	**0.51**	10.99

U343MG-a		Concomitant TMZ (250 *μ*M)			Sequential TMZ (250 *μ*M)		
DHMEQ (*μ*g/mL)	AF	AF	CI	DRI	AF	CI	DRI

2.5	0.08	0.37	**0.8**	1.56	0.26	1.17	1.08
5	0.10	0.44	**0.78**	1.92	0.41	**0.86**	1.76
10	0.22	0.58	**0.69**	2.86	0.58	**0.69**	2.86
20	0.53	0.79	**0.47**	5.84	0.80	**0.45**	6.10

U138MG		Concomitant TMZ (250 *μ*M)			Sequential TMZ (250 *μ*M)		
DHMEQ (*μ*g/mL)	AF	AF	CI	DRI	AF	CI	DRI

2.5	0.01	0.01	4.22	0.31	0.18	**0.48**	2.07
5	0.01	0.01	2.08	0.48	0.26	**0.36**	2.76
10	0.03	0.02	1.60	0.62	0.34	**0.28**	3.50
20	0.02	0.02	1.10	3.03	0.85	**0.06**	15.25

U87MG		Concomitant TMZ (250 *μ*M)			Sequential TMZ (250 *μ*M)		
DHMEQ (*μ*g/mL)	AF	AF	CI	DRI	AF	CI	DRI

2.5	0.00	0.53	**0.3**	7.1	0.42	**0.61**	2.42
5	0.10	0.45	**0.68**	3.26	0.61	**0.34**	15.71
10	0.27	0.60	**0.64**	14.2	0.83	**0.33**	248.40
20	0.69	0.88	**0.55**	666.16	0.94	**0.38**	4197.79

T98G		Concomitant TMZ (250 *μ*M)			Sequential TMZ (250 *μ*M)		
DHMEQ (*μ*g/mL)	AF	AF	CI	DRI	AF	CI	DRI

2.5	0.05	0.02	53.87	0.01	0.26	**1.00**	1.20
5	0.11	0.08	7.58	0.15	0.36	**0.70**	2.39
10	0.18	0.13	4.52	0.6	0.39	**0.85**	2.88
20	0.43	0.32	1.84	0.76	0.82	**0.22**	50.10

LN319		Concomitant TMZ (250 *μ*M)			Sequential TMZ (250 *μ*M)		
DHMEQ (*μ*g/mL)	AF	AF	CI	DRI	AF	CI	DRI

2.5	0.09	0.41	**0.37**	4.37	0.44	**0.33**	5.1
5	0.25	0.55	**0.3**	8.90	0.34	**0.68**	2.74
10	0.44	0.7	**0.27**	20.09	0.89	**0.09**	96.11
20	0.67	0.8	**0.31**	39.6	0.88	**0.19**	84.93

**Table 3:** Effects of DHMEQ on the radiosensitivity of GBM cell lines. Single cells were pretreated with 10 *μ*g/mL for 24 h and irradiated with 2, 4, and 6 Gy. After 7 days survival fractions were calculated. Dose enhancement rate (DER) >1 denotes a supraadditive effect. § Colony formation capacity was completely abolished after combined treatment.

		Cell line					
		U251	U343MG-a	U138MG	U87MG	T98G	LN319
DHMEQ 10 *μ*g/mL +	2 Gy	5.50	3.29	17.00	0.95	3.00	4.73
	4 Gy	9.50	8.50	16.36	1.30	2.50	4.47
	6 Gy	§	8.00	1.33	1.35	3.75	3.47
